# Green Management of Postharvest Anthracnose Caused by *Colletotrichum gloeosporioides*

**DOI:** 10.3390/jof9060623

**Published:** 2023-05-28

**Authors:** Yeimmy Peralta-Ruiz, Chiara Rossi, Carlos David Grande-Tovar, Clemencia Chaves-López

**Affiliations:** 1 Programa de Ingeniería Agroindustrial, Facultad de Ingeniería, Universidad del Atlántico, Puerto Colombia 081008, Colombia; 2 Faculty of Bioscience and Technology for Food, Agriculture and Environment, University of Teramo, 64100 Teramo, Italy; crossi@unite.it; 3 Grupo de Investigación de Fotoquímica y Fotobiología, Universidad del Atlántico, Carrera 30 Número 8-49, Puerto Colombia 081008, Colombia; carlosgrande@mail.uniatlantico.edu.co

**Keywords:** anthracnose, biocontrol, climate change, cultivar resistance

## Abstract

Fruits and vegetables are constantly affected by postharvest diseases, of which anthracnose is one of the most severe and is caused by diverse *Colletotrichum* species, mainly *C. gloeosporioides*. In the last few decades, chemical fungicides have been the primary approach to anthracnose control. However, recent trends and regulations have sought to limit the use of these substances. Greener management includes a group of sustainable alternatives that use natural substances and microorganisms to control postharvest fungi. This comprehensive review of contemporary research presents various sustainable alternatives to *C. gloeosporioides* postharvest control in vitro and in situ, ranging from the use of biopolymers, essential oils, and antagonistic microorganisms to cultivar resistance. Strategies such as encapsulation, biofilms, coatings, compounds secreted, antibiotics, and lytic enzyme production by microorganisms are revised. Finally, the potential effects of climate change on *C. gloeosporioides* and anthracnose disease are explored. Greener management can provide a possible replacement for the conventional approach of using chemical fungicides for anthracnose postharvest control. It presents diverse methodologies that are not mutually exclusive and can be in tune with the needs and interests of new consumers and the environment. Overall, developing or using these alternatives has strong potential for improving sustainability and addressing the challenges generated by climate change.

## 1. Introduction

Plant pathogens, such as *Colletotrichum gloeosporioides,* which cause the disease known as anthracnose, can attack vegetables and fruit crops both in the field and during the postharvest storage and commercialization phases, reducing their shelf life and generating the need to use pesticides, which are usually produced through chemical synthesis, as a disease mitigation strategy [1]. For several decades, this solution has been the most used solution for postharvest disease management; however, some studies have reported that chemical pesticides have adverse effects on the environment [2]. Additionally, consumer interest in high-quality, microbiologically safe, and sustainable products, together with initiatives approved by the United Nations (UN) known as the 2030 Agenda for Sustainable Development, has increased the interest in seeking new alternatives to plant and food protection [3]. Several technologies have been used to extend the shelf life of fruits during the postharvest stage, including freezing, sterilization, ozone, ethylene pretreatment, modified atmosphere, and heat treatments [4]. Although these technologies are effective, there are still some disadvantages to their application, including cost and changes in the physicochemical and sensory properties of the food products [5].

The use of natural substances has gained relevance, and countless studies have shown their advantages, including the postharvest conservation of fruits’ physicochemical and sensory qualities for a longer time. The most common materials used are biopolymers, including polysaccharides, proteins, and fats. In recent years, a wide variety of extracts and essential oils have been studied for their antimicrobial, antioxidant, insecticidal, and herbicidal activity, and the films and coatings prepared with the substances mentioned above are generally nontoxic and biodegradable [6]. Another up-and-coming green alternative is the so-called biological control, where residential or introduced biotic organisms are used to minimize the activities and population of plant pathogens [7]. These organisms include bacteria, fungi, viruses, protozoans, and insects, which can control plant diseases directly or indirectly [8]. Biocontrol also includes nonliving agents of biological origin and the compounds secreted by several organisms.

## 2. *Colletotrichum gloeosporioides*

*Colletotrichum gloeosporioides* is a cosmopolitan pathogen widely disseminated as a common plant pathogen globally [9]. It was first isolated from citrus in Italy and proposed by Penzig (1882) as *Vermicularia gloeosporioides*. The fungus includes *Glomerella cingulata* as a sexual teleomorph state (perfect) and *C. gloeosporioides* as an anamorph state (imperfect), and this concept was taxonomically formalized in 1957 [10].

The *Colletotrichum gloeosporioides* complex is one of the major clades of the genus *Colletotrichum*, comprising more than 22 species to date. The characterization of this fungus has mainly been based on its production of oblong to ovoid, hyaline, obtuse-ended conidia, as well as colony color, growth rates, appressoria shape [11], host type, presence of setae, whether or not the teleomorph develops, and the use of internal transcribed spacer (ITS) sequence [12]. However, some challenges and problems have been presented due to the variability of its morphological characteristics. This variability depends on the culture medium, growth conditions, difficulty in standardization, variable host range, pathogenicity, and erroneous sequence names of *Colletotrichum* strains in public databases generating deficient identification in delimiting species of *Colletotrichum* [13].

Molecular techniques have been used to resolve systematic problems in *Colletotrichum* species; in particular, the multilocus phylogenetic analyses with the uses of seven housekeeping genes [14], such as glutamine synthetase (GS), glyceraldehyde-3-phosphate dehydrogenase (GAPDH), calmodulin (CAL), actin (ACT), chitin synthase (CHS–1), β-tubulin (TUB2), DNA lyase (APN2), and mating-type protein (MAT1-2) genes that show high resolution to distinguish species in the *Colletotrichum gloeosporioides* complex [15]. Currently, the multilocus phylogenetic analyses are used together with the pathogenicity test, morphological description, and ecological and metabolite studies to accurately identify *C. gloeosporioides* [16].

### Colletotrichum gloeosporioides Infection Process

**Figure 1:**
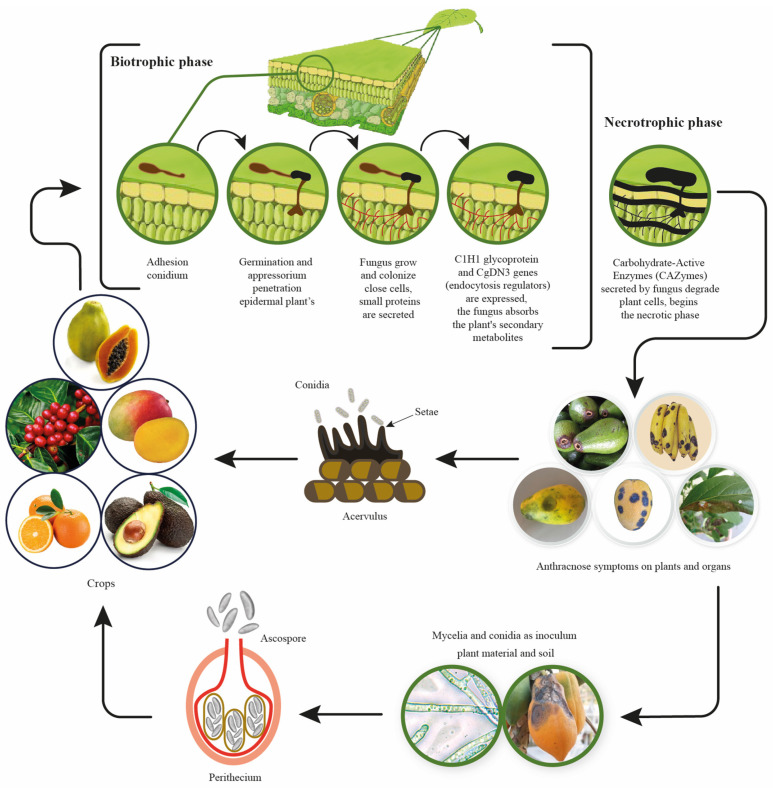
*Colletotrichum gloeosporioides* infection process and life cycle based on information by Siddiqui [17].

The *Colletotrichum gloeosporioides* initiates plant infection in a biotrophic manner (Figure 1), establishing itself in the plant after conidial adhesion to the leaf surface and subsequent spore germination. The fungus then penetrates the plant’s epidermal cells through an appressorium following the development of a small vesicle [17]. Primary hyphae grow and colonize only select cells, while the fungus secrets small proteins and absorbs secondary metabolites from the plant through its interaction with the apoplast. During this stage, genes critical to endocytosis, such as C1H1 glycoprotein and CgDN3, are expressed by the fungus [18]. The necrotic phase is characterized by the appearance of disease symptoms in the plant; the fungus produces secondary hyphae that ramify through host tissues and release carbohydrate-active enzymes (CAZymes), such as polygalacturonase and pectate lyase, which degrade plant cells and reduce host defense responses due to the high activity of oligogalacturonides that are released [17].

## 3. Anthracnose: The Big Problem with *Colletotrichum gloeosporioides*

Anthracnose disease is associated with significant economic losses of fruit and vegetable crops and evergreen trees and shrubs due to its incidence in preharvest and postharvest stages, affecting many crops globally, mainly in tropical and subtropical climates. It is caused by diverse species of *Colletotrichum* such as *C. coccodes, C. fructicola, C. siamense, C. capsici, C. gloeosporioides, C. acutatum, C. dematium, C. panacicola, C. boninense*, and *C. godetiae*. However, *C. gloeosporioides* has been identified as the prevalent cause of anthracnose [17].

*Colletotrichum* spp. infects fruits and other plant organs, including the leaves, flowers, twigs, and branches. It has been reported that the fungus can present an endophyte behavior and remain quiescent, infecting the plants without showing any symptoms [17]. Anthracnose causes the wilting and drying of tissues. Infected plants develop dark-colored spots on their leaves, as well as browning, curling, cupping (in the young leaves), and early drop. The above symptoms can also occur in flowers. In the preharvest stage, the fruit can present small, rounded spots on the surface; however, the fungus can remain dormant until the postharvest as appressorium, developing dark spots, decay, and rot in the fruit until the postharvest [18]. It is well known that some species of *Colletotrichum* infect specific hosts, while others infect multiple hosts. Anthracnose pathogens that infect multiple hosts may indicate the development of cross-infection ability. For example, isolates of *C. gloeosporioides* sensu lato from mango could infect and produce symptoms in guava, chili, and papaya [19]. 

**Figure 2:**
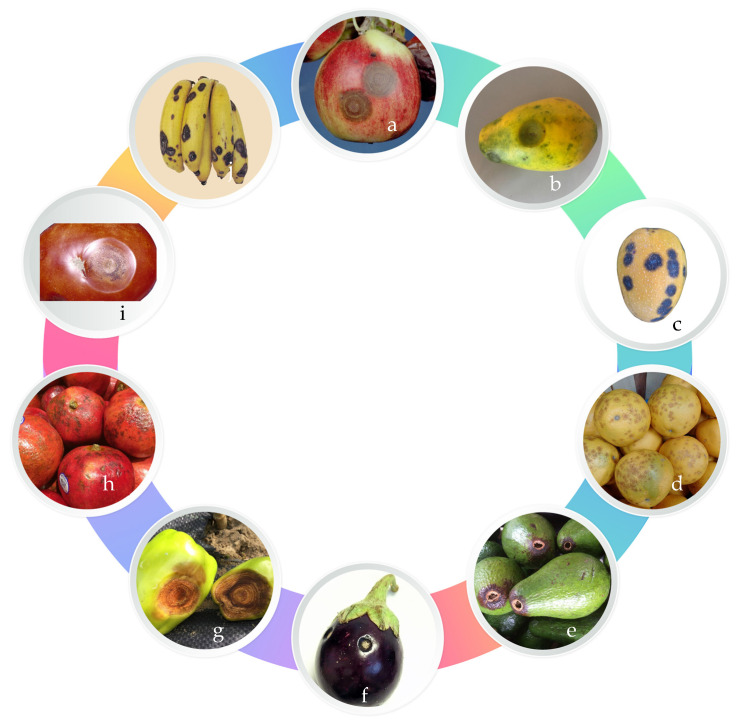
Anthracnose lesion caused by *Colletotrichum. gloeosporioides* in ripened fruits. (**a**) Apple, (**b**) papaya, (**c**) mango, (**d**) orange, (**e**) avocado, (**f**) eggplant, (**g**) pepper, (**h**) pomegranate, (**i**) tomato, and (**j**) banana. The lesions appear on the fruit as dark spots, gangrene, and rots. Source: By authors.

The environmental conditions that favor anthracnose disease are relative humidity greater than 90% and temperatures between 22 and 32 °C. These conditions are where most symptoms develop [10]. However, during postharvest, tissue damage can occur in drier conditions due to the presence of the fungus and the aging of the fruits. The pathogen can survive in crop residues and grow saprotrophically after being dispersed by rain splashes and air transmission [20]. Anthracnose has been reported in different hosts, affecting plant productivity and products such as mango, papaya, olive, coffee, banana, eggplant, and guava, among others (Figure 2).

### Colletotrichum gloeosporioides, a Globally Distributed Fungus

**Figure 3:**
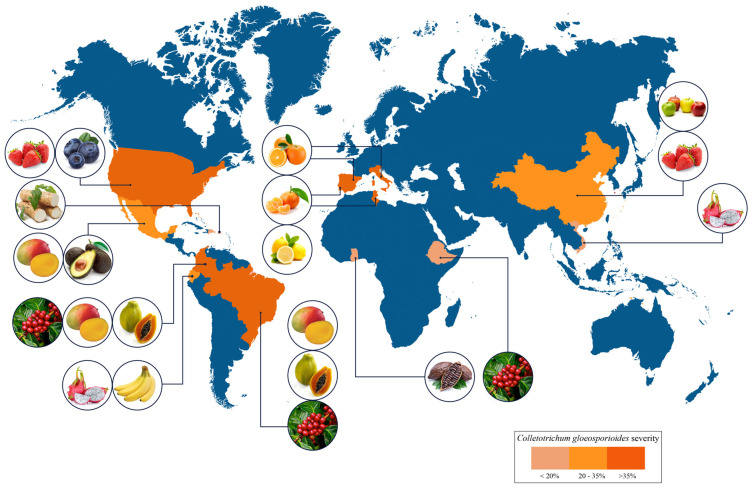
Worldwide distribution, occurrence, and severity of anthracnose. Source: By authors.

Anthracnose affects several fruits worldwide, mainly those produced in tropical and subtropical regions (Figure 3). High damage incidence caused by *C. gloeosporioides* has been reported in many countries including India, China [21], Indonesia, Mexico, Brazil, Ecuador, Guatemala [19], Belize, Costa Rica [22], Peru [23], Dominican Republic [24], Colombia [13], Australia [25], Italy [26], New Zeland [27], United States [28], and South Africa [19], among others.

Tropical fruits are susceptible to anthracnose disease both preharvest and postharvest [19]. This disease is one of the causes of the papaya shelf-life limitations, resulting in significant losses in the postharvest stage of around 25% to 40% [29] of the 13 megatons produced globally [30]. In the last decade, anthracnose has severely affected avocado production in Mexico, reducing fruit quality and commercial yield [31]. Similarly, mango fruit, one of the most important tropical fruits globally with a production of 58 megatons in 2019 [32], experienced direct and indirect losses in pre- and postharvest stages caused by anthracnose up to 30% in 2019 [33,34].

Recently, it has been reported that *C. gloeosporioides* causes the presence of anthracnose in bananas (Musa AAA Cavendish), the most crucial crop in Ecuador [19], as well as in blueberry crops in the United States [35]. Regarding citrus, Italy and Portugal have reported anthracnose disease in orange fruits, mandarin leaves, and unripe and ripe lemon fruits, respectively [26].

In 2019, Ghana reported economic losses from an anthracnose outbreak in cacao crops, the main export product [36]. In addition, coffee anthracnose is widely distributed in coffee-growing countries globally, causing devastating damage and significant crop losses [37]. The most harmful yam pathogen is *C. gloeosporioides*, which has reduced the production yield in Africa, the Caribbean, Asia, and South Pacific regions; in countries such as Puerto Rico, it has reported losses of 250 tones due to anthracnose disease [38,39]. Emerging super crops such as dragon fruit, highly desired for their nutraceuticals and functional properties and cultivated in many countries, including China, Colombia, Ecuador, and Vietnam, are affected by anthracnose, reducing their quality and decreasing the opportunity for export [19]. Anthracnose also presents difficulties for dried fruit; China has reported walnut production problems caused by fruit gangrene, which is caused by *C. gloeosporioides* [21]. On the other hand, the disease also attacks popular and globally produced fruit such as strawberries. The United States and China are reported as the principal causal agent of the strawberry anthracnose caused by *C. gloeosporioides* [28].

## 4. Climate Change (CC) and Its Probable Effects on *C. gloeosporioides* and Anthracnose Disease

It is well established that environmental changes, such as increasing temperature, light and water availability, soil fertility, wind speeds, atmospheric ozone, methane, CO_2_ concentrations, and changing precipitation, are linked directly to changes in plant-pathogen incidence and severity [40]. Thus, climate change will probably alter plant diseases’ geographical and temporal distribution; therefore, new problems will emerge, while other problems will be reduced. However, it is essential to consider how plants and pathogens adapt to changing environmental conditions and how the interaction could be harmful in the new climatic scenario. In this context, there is very little information about host and pathogen adaptation to climatic change, and accurate predictive models still do not exist for many diseases [41]. 

Meteorological parameters that predict crop disease occurrence include air temperature, leaf wetness, precipitation, and relative humidity. As mentioned above, the *Colletotrichum* growth is favored at temperatures and relative humidity above 20 °C and 95%, respectively. Guyader [42] reported the importance of preserving the genetic diversity of *C. gloeosporioides* strains since some strains/races have better resilience than others. *Colletotrichum* gloeosporioides spores can survive in leaf wetness absence for up to 48 h, and infectious capacity is recovered after high-temperature stress of up to 40 °C, depending on the strain. Misra [43], suggested that the decreasing rainfall resulting in lower humidity will also be unfavorable for the anthracnose disease of mango. 

The last decades have seen a dramatic increase in atmospheric CO_2_ concentrations, which is one of the major concerns for crop production in the face of CC. Some studies have demonstrated that increasing CO_2_ concentrations could increase crop yield; however, it might modulate host–pathogen interaction and related disease epidemics differently [44]. For the fungal pathogen *C. gloeosporioides*, it has been reported that an elevated CO_2_ concentration resulted in a modulation of the development and disease severity, which is highly dependent on the variety of the plant host and CO_2_ concentration. For example, Chakraborty et al. [45] reported that 700 ppm of CO_2_ delayed and reduced germination, germ tube growth, and appressoria production of *Colletotrichum*, thus decreasing the anthracnose severity. Changes in CO_2_ concentration from 700 ppm to ambient exposure influenced the susceptibility of the host (Stylosanthes scabra) under field conditions. In addition, aggressiveness has increased towards the resistant one but not the susceptible one.

Further studies have shown that the genetic fingerprint and karyotype of *C. gloeosporioides* isolates changed for some CO_2_ cultivar combinations but these changes were not related to increased aggressiveness [45]. Additionally, Cogliati et al. [46] demonstrated that a combination of 800 ppm CO_2_ and temperature between 22 and 26 °C resulted in a significant increase in anthracnose disease in terms of the percentage of affected leaves of plants, among other environmental conditions. However, increased levels of CO_2_ can decrease the susceptibility of coffee to *C. gloeosporioides* due to the high concentration of endogenous caffeine, which is reduced under high CO_2_ concentrations. Although the studies did not consider the combined effect with the temperature changes, these results are relevant not only to the genetics of *C. gloeosporioides*–host interaction but also to the epidemiology of the disease because it is the demonstration that the pathogen can adapt to a new environment; thus, it may make the expansion of the geographic area of *C. gloeosporioides* more concerning. Instead, three years of study by Koo et al. [47] showed that the aggressiveness of the anthracnose pathogen in hot pepper was significantly reduced in environmental conditions of 700 ppm CO_2_ and response to a temperature increase of 5 °C after 100 inoculation cycles. Studies have shown that increasing CO_2_ concentrations could boost crop yield; however, it might modulate host–pathogen interaction and the related disease epidemics differently. Li et al. [48] elevated the concentration of CO_2_-reduced caffeine (an alkaloid in tea that has long been known for its role in plant defense) and jasmonic acid (a crucial signaling molecule in plant defense) concentration, sharply increasing the susceptibility of tea to *C. gloeosporioides*. In addition, it should be noted that enlarged plant-canopy architecture under elevated CO_2_ can trap more spores, leading to more severe anthracnose under favorable weather. 

Finally, CC could reduce the effectiveness of biological control agents for *C. gloeosporioides*, which could be a significant problem in future pest-management programs. CC causes a considerable reduction in the yield of major crops worldwide and food security. However, how the combined effects of multiple environmental conditions impact disease outcomes remains one of the most outstanding and challenging questions for the future study of plant–pathogen interactions [40]. The studies mentioned above did not consider the coevolutionary processes of *Colletotrichum* spp. with superior plant varieties, fungicides, and agricultural practices.

## 5. Anthracnose Management: New Green Alternatives

Maintaining good sanitary conditions in fruit storage is essential to minimize postharvest contamination by pathogenic microorganisms. Anthracnose management in preharvest and postharvest fruits and vegetables includes fungicides such as benomyl, mancozeb, propineb, prochloraz, metiram, and copper oxychloride, and thiabendazol, commonly used to control anthracnose [49]. However, their use may be subject to internal regulations in each country. 

The use of biocontrol agents such as biocoatings and biofilms, reinforced or not with extracts and essential oils, and the use of antagonistic microorganisms, have been reported in several investigations in recent years, following the concept of sustainability for anthracnose postharvest management [9,29].

### 5.1. Essential Oils

Essential oils (EOs) are complex mixtures mainly of low molecular weight terpenic compounds, soluble in nonpolar solvents, less dense than water, and characterized by being volatile with a strong fragrance [50]. Essential oils are considered generally safe natural food preservatives (GRAS) for consumption [51] unless they negatively affect the sensory and organoleptic qualities of the food or show some sign of toxicity that should always be checked. Due to their vast insecticidal, herbicidal, bactericidal, and fungicidal capacity, low toxicity for humans, and harmlessness to the environment, the application of essential oils in agriculture has grown considerably in recent years as an alternative to synthetic pesticides for crop protection [50]. 

Recent review articles have reported fascinating results on the use of essential oils from aromatic plants to control *Colletotrichum* species [51]. However, many aspects related to the antifungal activity of the oils on fungi of the genus *Colletotrichum*, such as the mechanism of action, the individual effect of the main components, and the phytotoxicity of these oils with other species, remain unresolved. Therefore, it is crucial to continue growing the number of investigations of essential oils against these fungi, which cause diseases in crops.

Several studies have addressed the use of essential oils for *C. gloeosporioides* control. Table 1 shows examples of the last six years of applying essential oils to control *C. gloeosporioides* in vitro and in situ. For example, Sarkhosh et al. [52] tested the inhibitory effect of essential oils from Thyme (Thymus daenensis Celak.), savory (Satureja khuzistanica Jamzad.), mint (Mentha piperita Willd.), cinnamon (Cinnamomum zeylanicum Blume.), and lavender (Lavandula angustifolia Mill.) both in vitro and in situ. The essential oils of savory and thyme were the most effective in controlling the growth of *C. gloeosporioides* with 100% inhibition of mycelial growth under in vitro conditions and an EC_50_ = 58.42 µL L^−1^ (for the Savory oil). In situ, an oil concentration of 2000 µL L^−1^ decreased 59.26% and 58.40% lesion diameters in papayas that maintained their firmness better than the other applied oils. The reported EC_50_ for the savory oil in papayas was 1507.19 µL L^−1^, making this oil promising as a natural antifungal agent.

Despite the advantages of using essential oils to protect crops and reduce the decay of fruits and vegetables, their high volatility and the need to use high concentrations somewhat restrict essential oils for food protection. For this reason, essential oils are combined with biodegradable polymers that facilitate their controlled release, improve their antimicrobial activity, and allow the use of low concentrations as coatings on fruits to prevent their decay [9].

### 5.2. Plant Extracts

The plant extracts possess antimicrobial activity and have a promising role as environmentally friendly agents as an alternative to synthetic fungicides in controlling *C. gloeosporioides*. Several studies have reported the antifungal activity of plant extracts both in vitro against *C. gloeosporioides*, and in situ for managing postharvest anthracnose of fruits and vegetables.

Regarding in vitro studies, plant extracts from Aegle marmelos, Ipomoea carnea, and Pongamia pinnata at a concentration of 15% inhibited the mycelia growth of *C. gloeosporioides* by 74.85%, 63.23%, and 54.89%, respectively [66]. In another screening, Kwodaga et al. [67] reported that a 100% aqueous extract of Capsicum annum fruit inhibited *C. gloeosporioides* mycelial growth and spore germination by 76.1% and 91.7%, respectively. España et al. [68] detected more potent *C. gloeosporioides* inhibition using ethanolic extracts than essential oils from Eucalyptus species. In particular, the most active extract was E. camaldulensis (5000 mg/L), which showed 98% inhibition of fungal growth. Moreover, the ethanolic extract of E. globulus showed a half reduction of *C. gloeosporioides* growth at low concentrations (500 mg/L). 

Most investigations have been performed using crude extracts to test the synergistic effect of mixtures with solvents. Several studies have compared the efficacy of plant extracts obtained through different extraction procedures using different alcohols, such as ethanol and methanol, as organic solvents. Bussaman et al. [69] showed that methanol, chloroform, and 80% ethanol extracts of Piper sarmentosum leaves effectively inhibited the mycelium growth of *C. gloeosporioides* by 100%, 81.85%, and 45.50%, respectively. Among these three extracts, the total inhibition of fungal-spore germination was obtained with crude methanol extract at 1.25% concentration. The methanol extract of Azadirachta indica, a versatile tree of the family Meliaceae known as neem, is also effective as an antifungal agent, inhibiting *C. gloeosporioides* growth by 37% and by 14% with acetone and water extracts, respectively [70].

In a different study [71], it was found that acetone, methanol, and aqueous extracts of Thevetia peruviana inhibited the growth of *C. gloeosporioides*, with inhibition percentages ranging from 11.90% to 93%. The acetone extract at a concentration of 6.25 µL/mL completely inhibited the spore germination of all the tested strains, and its effectiveness was confirmed in situ on cassava, where it reduced the severity of the disease. 

Regarding in situ studies, an aqueous extract of Plantago sinaica also inhibited the growth of *C. gloeosporioides* both in vitro and on artificially inoculated mango, where the severity of the disease was significantly reduced. The antifungal potential of the extract could probably be due to its high content of phenolic compounds [72]. De Guzman Alvindia and Mangoba [73] evaluated the effect of Allium longicuspis against mango anthracnose. Complete in vitro inhibition of *C. gloeosporioides* was found using the extract concentrated from 0.75 to 2.5 g/L. After the extract application, the authors observed clear cytoplasmic discharge and mycelial and conidial blasts. In addition, in situ results showed a reduction in anthracnose incidence on mango fruit after extract application before, after, and simultaneously with *C. gloeosporioides* inoculation. 

Marinho et al. [74] evaluated the soapberry leaf extract effect (100 mg/mL) on anthracnose development in papaya fruits, and the results showed a reduction in anthracnose symptoms in the tested fruits. Among other plants, ginger, turmeric rhizome, and “dukung anak” crude extracts were found to possess antifungal activity against *C. gloeosporioides* growth and conidial germination, causing distortion, shrinking, and swelling of fungal hyphae [75]. Additionally, an in vitro study showed that the dukung anak and turmeric crude extract (5 and 10 g/L concentrations) reduced anthracnose infection levels in dragon fruit. However, when the plant crude extracts were used at 15.0 g/L, they compounded disease incidence (DI) and disease severity (DS) due to phytotoxicity. 

To improve the storage quality of fruits and control fungal diseases, researchers have explored the use of edible coatings containing plant extracts as novel postharvest treatments. Tesfay et al. [76] reported that using a carboxymethyl cellulose edible coating combined with moringa leaf and seed extracts prolonged the shelf life of avocados. The authors demonstrated that the coating treatments reduced the incidence and severity of anthracnose. Scanning electron microscopy images revealed that the moringa extracts caused morphological changes in *C. gloeosporioides* hyphae, such as shrinkage, broken strands, and constriction.

The potential of plant extracts to inhibit anthracnose disease in fruits, as demonstrated by various studies, presents an opportunity to develop innovative solutions for controlling *C. gloeosporioides* in the agri-food industry. However, further research is necessary to better understand the mechanisms of action of these extracts in vitro and their effectiveness in situ, particularly when used in combination with other ecologically-friendly approaches.

### 5.3. Biodegradable Polymers

#### 5.3.1. Chitosan

Chitosan is a polymer that results from the deacetylation of at least 50% of chitin, a polysaccharide abundant in nature, found in crustaceans, insects, and the mycelium of many fungi [6]. Chitosan and its synthetic derivatives are more soluble in dilute acid solutions than chitin, making it easier to process. It has many beneficial properties, such as biocompatibility, elasticity, adhesiveness, chelating capacity for anions in solution, biodegradability, adsorption, prebiotic capacity, and antioxidant capacity, among others, which have attracted much attention for various applications in medicine, agriculture, cosmetics, pharmaceuticals, water treatment, and the food industry [77].

One of the most prominent applications of chitosan in the food industry is as a polymeric matrix used to form films and coatings that extend the shelf life of food [6]. However, chitosan applied as a coating or packaging material suffers from significant drawbacks, including low resistance to tension, high water permeability, and brittleness, among others. These factors require attention for efficient application in food preservation. To improve these properties, additives such as lipids, waxes [78], or clays [79] can be added to increase the hydrophobicity and elasticity of the films, although this may affect mechanical and organoleptic properties. These issues have been partially resolved by adding essential oils (EOs) [6]. 

#### 5.3.2. Chitosan Edible Coatings

Edible films and coatings are thin layers of edible materials, mainly biopolymers, applied to the surface of foods to improve stability and promote preservation and acceptance by consumers. The coatings introduce barrier, antimicrobial, and antioxidant properties to the food surface [80].

Natural polymers and their (nano)composites, such as proteins and polysaccharides, are used to create biodegradable, edible coatings. These materials are highly organized with packed supramolecular structures that utilize hydrogen bonds and other noncovalent interactions to block the loss of volatile aromatic metabolites, reduce dehydration and loss of firmness, and decrease enzymatic browning by regulating the introduction of oxygen. This preservation allows the organoleptic characteristics of food to be maintained for longer periods [80]. Recently, chitosan has received much attention in the food industry as a GRAS compound [6]. However, the problems inherent to the processability of polysaccharides are not absent in chitosan, which is why it is necessary to prepare composites or use nanofillers to prepare coatings with better properties and performance [81].

On the other hand, chitosan nanoparticles have been reported to be effective in controlling the infection caused by phytopathogenic fungi such as *Botrytis cinerea*, *Rhizopus stolonifer*, and *Aspergillus niger* in strawberries under both in vivo and in vitro conditions. The nanoparticles produced had an average size of 331.1 nm (±7.21) with a narrow polydispersity index (0.377) and a zeta potential of +34 mV, which demonstrated the cationic character of the nanoparticles that contributed to reducing fungal infection by damaging cell morphology [82].

Additionally, chitosan has been emulsified with essential oils to introduce hydrophobicity, antioxidant, antibacterial, antifungal, insecticidal, and other biological properties of broad interest [9]. In the coming years, it is expected that research on (nano) composites of chitosan and essential oils will continue to grow, thanks to the renewed interest in more environmentally friendly green chemistry products and the exciting properties that are being introduced with the essential oils mentioned above. 

Concerning *C. gloeosporioides* control, films and coatings have been designed for the anthracnose control developed in various fruits. These studies report inhibition under in vitro conditions and directly inoculated fruits, as shown in Table 2.

### 5.4. Other Polymers

Biodegradable polymers, in addition to chitosan, have been tested for anthracnose control. Polymers such as aloe vera gel (consisting mainly of polysaccharides), methylcellulose, starch, and gum arabic have been shown to suppress fungal decay, helping to maintain the quality of the fruit [86]. Mendy et al. [87] reported the effect of fresh and food-grade aloe vera gel aqueous solutions in concentrations of 15%, 25%, and 50% (*v*/*v*) on different pathogens responsible for fungal decay of papaya fruit, including *C. gloeosporioides*, both in vitro and in situ. The study showed inhibition of 72.96% of the fungus in vitro. Aloe vera gel showed a dose-response behavior in situ studies on papaya; the fungus spores were artificially inoculated, and after 72 h at room temperature, total *C. gloeosporioides* growth inhibition was evident. Some studies have reported the effect of aloe vera gel in combination with other polymers, essential oils, and proteins. Bill et al. [86] observed that aloe vera reinforced with thyme oil coatings (1:1 or 3:1 *v*/*v*) presented a fungicidal effect against *C. gloeosporioides*. However, aloe vera alone showed a fungistatic effect. In situ tests on avocados showed a reduction of 58.6% and 40% in anthracnose severity with aloe vera–thyme oil and aloe vera coatings, respectively. In addition, an increase in defense response-related enzymes in avocados was observed.

Starch has presented promising results in anthracnose control. Cassava starch coatings in 1–4% concentrations were tested on papaya inoculated with *C. gloeosporioides* conidia; with 2–4% of cassava starch, total disease inhibition and reduced fruit maturation were observed. The arrowroot starch edible coatings in vitro effect reinforced with *Piper aduncum* essential oil was evaluated against *C. gloeosporioides*; these coatings showed antifungal activity with the concentrations of 0.75 and 1% of EO being dose-dependent; the coatings also presented good barrier properties [88].

On the other hand, Sousa et al. [89] studied in situ edible coatings of hydroxypropyl methylcellulose (HM) and beeswax (BW) on mango. The HM+ 40% BW coatings showed a reduced anthracnose incidence of 66%, while also maintaining the color of the peel and delaying fruit ripening. Similar results were reported by Vieira et al. [90] who used HM with 0.25% silver nanoparticles to control anthracnose in papaya and extend its shelf life. 

Gum arabic has also been tested as a microencapsulating agent for controlling *C. gloeosporioides*. Microcapsules of gum arabic with thyme oil (3:1 *w*/*w*) showed activity against the fungus in vitro, with a slight growth rate. The antifungal effect was also evaluated over time, with the microcapsules maintaining their effect almost constantly for up to six days [91].

### 5.5. Proteins

The use of protein agri–industrial waste to produce bioactive peptide compounds, which have been demonstrated to have antimicrobial and antioxidant activities [92], has become an attractive alternative for disease control. It has been reported that cottonseed protein hydrolysates films showed an inhibitory effect against *C. gloeosporioides* with an inhibition zone of 27.02 mm and cottonseed byproduct liquid hydrolysate, with growth inhibition of 70.78% [93]. Garcia. [94] evaluated the effect of corn zein nanoparticles against two *Colletotrichum* species both in vitro and inoculated in avocado, reporting a reduction in the growth of 34.1 and 50% for concentrations of 1000 ppm and 500 ppm, respectively, with a consequent reduction in fungal decay. 

In the last year, interest in new protein sources with biotechnological applications has increased, where endemic plants of the Amazon region have demonstrated a high potential. *Cereus jamacaru* (Brazilian plant) protein root and stem extract showed an inhibition effect in *C. gloeosporioides* of 43%. This inhibition was accompanied by morphological changes in the fungus with increased fungal membrane permeability, reactive oxygen species (ROS) induction, and cell death [95]. On the other hand, protein extracted from *Clitoria ternatea* seed, a tropical forage legume known for its high resistance to pathogens and pests, inhibited the *C. gloeosporioides* growth of its high concentration of the protein that authors denominated ‘finotin’, this protein also present activity against other filamentous fungi [96].

The antimicrobial activity of proteins can also be enhanced by using nanoparticles (NPs), which are promising solutions. Nguyen et al. [97] tested gelatin coatings with silver NPs for the control of *C. gloeosporioides* in vitro and in situ on mangoes. They observed a complete inhibition of fungal growth on Petri dishes but did not find significant inhibition on mangoes compared to the control.

### 5.6. Bacteria and Fungi as Biocontrol Agents for the Management of Postharvest Anthracnose

Biological approaches to disease control have gained popularity, and research in this field has intensified in recent years. Microbial antagonists such as bacteria and fungi have been found to be effective against phytopathogenic fungi. These microorganisms can be isolated from the fruit surface or lesions. Antagonists that are native to the environment where they are used have an advantage in biocontrol. However, they have also been isolated from other sources such as the phyllosphere, roots, and soil [98]. In recent years, studies have focused on marine yeasts and bacteria as an alternative for controlling postharvest decay in fruits caused by phytopathogenic fungi [99]. Table 3 provides a representative sample of studies on *C. gloeosporioides* biocontrol using bacteria and fungi on fruits and vegetables. In general, many studies have reported higher antifungal activity when biocontrol agents were applied before infection rather than postinfection [100].

Studies on the *C. gloeosporioides* control using bacteria were carried out in fruits such as apple, avocado, chili, loquat, and mango. *Bacillus* spp*., Stenotrophomonas* spp., *Streptomyces* spp., and several other species are promising biological control agents for managing *C. gloeosporioides.* Many *Bacillus* species were investigated as antagonistic microorganisms of *C. gloeosporioides*, such as *B. amyloliquefaciens*, *B. atrophaeus*, *B. licheniformis*, *B. mycoides*, *B. pumilus*, *B. subtilis*, *B. thuringiensis*, and *B. velezensis*. Among them, *B. amyloliquefaciens*, *B. pumilus*, and *B. thuringiensis* showed the highest inhibitory activity (>80%) in mango [98]. In addition, *Stenotrophomonas* spp. has been found to be effective for anthracnose control on mango [99], reducing the incidence and severity to 90%.

Biocontrol of *Colletotrichum gloeosporioides* using yeasts has been carried out in avocado, citrus, grape, mango, olive, papaya, chilly, apple, and rambutan (Table 3). Yeasts naturally present on fruit surfaces represent the primary group of yeasts utilized for postharvest disease control. In fact, Pesce et al. [101] demonstrated that most of the yeasts capable of controlling anthracnose caused by *C. gloeosporioides* in ripe olives (*Olea europea* L.) were isolated from the fruit surface (*C. albidus, W. anomalus,* and *P. kudriavzevii*) and closely related microenvironments (*C. tropicalis*, *W. anomalus*). 

However, yeasts isolated from a vitivinicultural environment (*P. membranaefaciens*, *S. chevalieri, T. delbrueckii*) have also shown activity against *C. gloeosporioides*. In the case of papaya, biocontrol has been studied using yeasts such as *Candida oleophila, Cryptococcus magnus, Meyerozyma guilliermondii*, and *Wickerhamomyces anomalus*. Additionally, *Debaryomyces hansenii* has been reported to have a strong capacity to reduce anthracnose disease incidence and severity by up to 100% in papaya and mango fruits [102]. 

Filamentous fungi have also been used for the management of *Colletotrichum* spp., mainly using *Trichoderma* species [103]. Formulations of antagonistic microorganisms have been proposed for commercial use after studying their capability for postharvest anthracnose control. Powder formulations of *Meyerozyma caribbica* have shown efficacy in managing postharvest on mangoes stored at 25 °C [104]. Additionally, researchers investigated the ability of *M. caribbica* combined with alginate to reduce anthracnose up to 100% in avocado fruit at 6 °C, while also reducing fruit weight loss and maintaining the viability of the antagonistic microorganism during storage [105].

#### 5.6.1. Mechanisms of Biocontrol

As shown in Table 3, antagonists can exert antifungal activity via numerous possible mechanisms, including competition for nutrients and space, biofilm formation, induction of host disease resistance, production of lytic enzymes, siderophores, antibiotics, and volatile organic compounds (VOCs).

##### Competition for Nutrients and Space

The capacity of antagonistic microorganisms to control fruit phytopathogenic fungi can be related to their competition for space and nutrients. It has been reported that when carbon sources decrease, microorganisms can rapidly consume them, thereby limiting the growth of phytopathogenic fungi [102]. Both yeasts and bacteria can reduce glucose, sucrose, and fructose, which limits spore germination of the fungus and consequently reduces infections in the host [106]. Tian et al. [107] found that *M. pulcherrima* competes effectively for nutrients, especially glucose, and can rapidly assimilate and absorb this essential carbohydrate. In addition, competition for space is one of the effective ways that the antagonistic yeast *M. pulcherrima* inhibits *C. gloeosporioides*. When *M. pulcherrima* is inoculated before the wounding of the mango, it can grow and develop rapidly, and the pathogens gradually die because there is no growing space.

##### Biofilms

To successfully colonize intact and injured fruit surfaces, the antagonist must have the ability to use specific features to facilitate its adherence, colonization, and multiplication [108]. Bautista-Rosales et al. [109] revealed that *C. laurentii* has high antagonistic potential against *C. gloeosporioides*, reducing lesion development in mango fruit by 75.88%. The yeast exhibited several mechanisms of action to manage mango anthracnose, including the ability to adhere and form biofilms on the fruit and *C. gloeosporioides* hyphae, showing competition for space. Studies have suggested that the process starts with cell adhesion to the host. Through chemical signals, extracellular polymeric substance (EPS) production, microcolonies formation, and biofilm formation, the pathogen growth is limited by nutrient competition in which yeasts are interposed between the pathogen and the substrate [109]. The results of Zhou et al. [110] showed that the action of *D. nepalensis* to control and inhibit the pathogen growth was to survive on lesions on the fruit surface and produce biofilm and adhere to the pathogens.

##### Induction of Host Disease Resistance

It is well established that enzymes related to the disease process in the plant are essential in the interaction between the host and pathogen by improving the resistance of the host to various stresses, such as disease, salt, and cooling [111]. Phenylalanine ammonia-lyase (PAL), catalase (CAT), and peroxidase (POD) have been suggested to be involved in host resistance mechanisms, protecting the plant against damage caused by pathogens. The induction of resistance may also be involved as a biocontrol mechanism by yeasts. Increased activities of defense enzymes are critical for *Diospyros nepalensis* to control *Colletotrichum gloeosporioides* [110]. In addition, Shao et al. [111] reported that the activity of PAL, polyphenol oxidase (PPO), chitinase (CHT), and β-1,3-glucanase (GUN) were enhanced in the fruits treated with *Metschnikowia pulcherrima* compared to the untreated fruits, suggesting that the inhibition of anthracnose disease with *M. pulcherrima* treatments may be due to accelerated activities of these defense enzymes during mango storage.

The microorganism’s enzymatic activity is considered an antagonistic mechanism; enzymes such as glucanase, protease, and chitinase are related to ß-glucan, mannoprotein, and chitin degradation, which are the main components of the fungi cell wall [102]. Reyes-Perez et al. [99] demonstrated the production of extracellular enzymes involved in fungal cell wall hydrolysis by *S. rhizophila*. For the same bacterial species, the detection of protease, ß-1,3-glucanase, and chitinase has also been reported by other authors [112]. In addition, proteases degrade the proteins within the pathogen cellular membrane, facilitating the yeast’s ability to mainly feed on a nitrogen and amino acid source [102].

##### ROS

Reactive oxygen species (ROS) are often detected in plant–pathogen interactions and are associated with the development of disease symptoms. Since CAT and POD are detoxifying enzymes capable of promoting ROS scavenging, the induction of their activities after treatment with *Pseudomonas putida* suggested that one of the mechanisms by which this bacterium inhibits fruit decay development could be the alleviation of pathogen-induced ROS [113]. In the study of Zhao et al. [114], the H_2_O_2_ and O_2_^−^ content was increased at the early stage of citrus fruit treatments with *P. membranaefaciens* and then was inhibited at the end stage. The enhancement of ROS content in the early period prevented their accumulation in the late period, probably due to the ROS acting as a secondary signal to induce defense responses.

##### Production of Antibiotics

Antibiosis is the phenomenon where antagonists suppress pathogen growth by producing compounds such as antibiotics [115]. Antibiotics of the iturin family are commonly studied for their activity, and among them, iturin A possesses a broad antifungal spectrum. Arrebola et al. [116] examined the role of *Bacillus amyloliquefaciens* isolated from citrus surfaces in postharvest pathogen control. The results proved the antagonistic strain’s capacity, which displayed the production of iturin A against *Colletotrichum gloeosporioides*. In addition, orange fruit trials confirmed disease inhibition when *B. amyloliquefaciens* was applied one day after *C. gloeosporioides* application. Iturin A increased cell membrane permeability and malondialdehyde (MDA) content, indicating that iturin A can effectively stimulate fungal oxidative stress, causing MDA to accumulate in the mycelium [117]. In addition, the authors found that protein content decreased in the *C. gloeosporioides* cell with the increase of iturin A concentration, which may be caused by the extravasation of cell contents or the failure of protein synthesis due to the destruction of organelle structures.

##### VOCs

VOCs are ideal antimicrobials and biofumigants because they do not require physical contact between antagonistic microorganisms and pathogens or between antagonistic microorganisms and the food product [118]. It has been reported that VOCs such as ketones, pyrazines, and sulfur-containing compounds produced by Bacillus could be responsible for *C. gloeosporioides* inhibition [24]. In addition, compounds produced by *Bacillus* species can activate antioxidant enzymes in the fruit (e.g., POD, PPO, CAT, and superoxide dismutase) to eliminate excessive ROS, thus reducing plant-cell damage. Reyes-Perez et al. [99] reported that the marine bacterium *S. rhizophila* inhibited *C. gloeosporioides* growth in vitro and on fruit by producing VOCs. VOC production by this bacterium has been identified as an antagonist mechanism, mainly by producing ß-phenyl ethanol and dodecanal [119]. Inhibition of phytopathogens through VOCs such as 2-methyl-1-propanol, 3-methyl-1-butanol, and 2-methyl-1-butanol has been reported for different yeasts, including *D. hansenii, W. anomalus*, *M. pulcherrima*, and *A. pullulans* [102,107]. These alcohols are absorbed in the phytopathogen cellular membrane increasing its permeability and accelerating the spreading of essential ions and metabolites through their membrane, inhibiting its growth and spore germination [102]. VOCs have been reported as being produced and released by several *Trichoderma* species; in particular, a total of 16 VOCs were detected in *T. Koningiopsis*, and the authors associated the suppression of *C. gloeosporioides* with the presence of azetidine, 2-phenyl ethanol, and ethyl hexadecanoate [103].

**Table 3:** A representative sample of in vivo studies of the biocontrol of *C. gloeosporioides* disease using bacteria and fungi.

Antagonist	Host/Test Plant	Mechanism of Biocontrol	Disease Incidence Inhibition	Disease Severity Inhibition	Lesion Length Inhibition	Reference
Bacteria						
*Bacillus* *amyloliquefaciens*	Strawberry (*Fragaria ananassa* cv Taoyuan)			≈50%		[120]
	Loquat (*Eriobotrya japonica* Lindl)	Iturin A				[117]
*Bacillus* *atrophaeus*	Soursop (*Annona muricata*)	Antibiotics	66%			[121]
	Avocado (*Persea americana*)	Antibiotics	40%			
*Bacillus* *Mycoides*	Avocado			42%		[24]
*Bacillus* *subtilis*	Chili (*Capsicum annuum* L. cv Arka Sweta)		80%			[122]
	Mango *(Mangifera indica* L. var. Tommy Atkins)				60%	[123]
	Mango *(Mangifera indica* L. var. Tommy Atkins)				58%	[123]
*Paenibacillus* *polymyxa*	Apple				≈60%	[124]
	Mango (*Mangifera indica* L. cv Ataulfo)	Competition for nutrients and space, lytic enzymes, siderophore, VOCs	95%		85%	[99]
*Streptomyces* *philanthi*	Chili (*Capsicum annuum* L.)	VOCs	33–100%			[125]
*Fungi*						
*Candida* *tropicalis*	Olive (*Olea europea* L.)		≈90%			[101]
*Cryptococcus* *albidus*	Olive (*Olea europea* L.)		≈76%			[101]
*Cryptococcus* *laurentii*	Mango (*Mangifera indica* L. cv Ataulfo)	Biofilms, competition for nutrients and space, induction of host disease resistance, lytic enzymes			75.88%	[109]
*Debaryomyces* *hansenii*	Papaya (*Carica papaya* L. var Maradol)	Competition for nutrients and space, lytic enzymes, VOCs	40–100%		66–100%	[102]
*Debaryomyces* *nepalensis*	Mango (*Mangifera indica* L. cv Tainong No. 1)	Biofilms, competition for nutrients and space, induction of host disease resistance, lytic enzyme, VOCs			4.7–93.4%	[110]
*Epicoccum* *dendrobii*	Apple	Metabolites			75–100%	[100]
*Metschnikowia* *pulcherrima*	Mango (*Mangifera indica* L. cv Tainong)	Induction of host disease resistance			≈40%	[111]
	Mango (*Mangifera indica* L. cv Tainong)	Competition for nutrients and space, induction of host disease resistance, VOCs, non-VOCs			55–100%	[107]
*Meyerozyma* *caribbica*	Mango (*Mangifera indica* L. cv Ataulfo)		53.4%		36.3%	[104]
*Papiliotrema* *aspenensis*	Mango fruit	Biofilms, competition for nutrients, siderophore, VOCs		94.1%		[118]
*Pichia* *membranaefaciens*	Citrus (*Citrus sinensis* L. Osbeck cv. Jincheng)	Cell membrane damage control, reduction of cell wall-related enzymes activity, ROS regulation	≈50%		≈67%	[114]
	Citrus (*Citrus sinensis* L. Osbeck cv. Jincheng)	Competition for nutrients and space, lytic enzymes	≈27%			[126]
*Saccharomyces* *chevalieri*	Olive (*Olea europea* L.)		≈50%			[101]
*Torulaspora* *delbrueckii*	Olive (*Olea europea* L.)		≈63%			
*Trichoderma* *koningiopsis*	Chili pepper	Competition for nutrients and space, lytic enzymes, VOCs			100%	[103]
*Trichoderma* spp.	Papaya (*Carica papaya* L. var Maradol)	Mycoparasitsm				[127]
*Wickerhamomyces* *anomalus*	Olive (*Olea europea* L.)		≈60–90%			[101]

### 5.7. Cultivar Resistance

Host-based immunity can effectively control diseases, but not all plants are naturally resistant to disease; as a result, disease management depends on the use of disease-resistant varieties. Using resistant varieties may eliminate losses from diseases as well as the chemical and mechanical expenses of disease control. The development of resistant varieties is possible via traditional breeding approaches or genetic engineering by introducing resistance mechanisms derived from other plant species or pathogens. However, creating and commercializing an anthracnose-resistant cultivar is challenging due to the diversity of the *Colletotrichum* species and pathotypes [128].

Some plant species, including chili (*Capsicum annuum* L.), strawberry (*Fragaria ananassa*), tomato (*Solanum lycopersicum)*, and walnut (*Juglans regia* L.), have been studied for the genetic regulations of disease resilience to develop resistant cultivars against anthracnose caused by *C. gloeosporioides*. One study identified six genotypes (Punjab Lal, Bhut Jolokia, BS-35, Pant C-1, CA-4, and Acchar Lanka) that have the potential to be used in chili breeding programs against anthracnose in India [128]. In addition, Mahto et al. [129] developed stable transgenic lines of chili and tomato expressing CgCOM1-RNAi construct employing Agrobacterium-mediated transformation. The fungal challenge assays on leaves and fruits showed that the transgenic lines were resistant to anthracnose disease-causing *C. gloeosporioides*. In strawberries, the octoploid progenitor species *Fragaria virginiana* and *F. chiloensis* have been utilized to improve cultivated strawberries [130]. Anciro et al. [131] described a locus, *FaRCg1*, which confers resistance against crown rot caused by *C. gloeosporioides* in cultivated strawberries. The identification and characterization of this locus have been used to create molecular tools that are used in breeding programs to improve genetic gains for resistance. Additionally, recent studies have provided experimental evidence that the JrWRKY21 gene can indirectly activate the expression of the JrPR5L gene via the WRKY21–PTI5L protein complex to promote resistance against *C. gloeosporioides* in walnuts [132].

The variable responses of mango cultivars to anthracnose disease across different locations, coupled with the fact that local evaluations of susceptibility have often failed to consider the specific *Colletotrichum* species responsible for infections, has hindered the consistent use of resistance as a means of controlling mango anthracnose [34].

Despite efforts to develop resistant cultivars through breeding programs, the rapid evolution of phytopathogens and the emergence of virulent forms that can overcome plant resistance render these cultivars quickly outdated. Breeders face challenges such as the use of a limited number of plants in breeding programs, the transfer of unwanted traits alongside useful resistance genes, and, more recently, the depletion of potential gene sources [133].

## 6. Conclusions and Prospects

In recent years, various strategies have been reported to manage anthracnose caused by *Colletotrichum gloeosporioides* [1,29,34,65]. A shift in consumer paradigms and their growing interest in healthy, organic foods and the wellbeing of the environment have led to the development of new green strategies that aim to minimize the use of chemical fungicides. Many publications have proposed the use of natural substances such as polymers, proteins, and phytocompounds such as essential oils, which have exhibited fungicidal activity against *C. gloeosporioides*. In addition to its activity against the fungus, its use individually or together prevents the deterioration of fruits, especially tropical ones, increasing their shelf life and preserving their physicochemical and sensory properties for longer. Biological control has also reported promising results, and studies have been directed to the search for new effective microorganisms against anthracnose. However, despite advances in these technologies, CC has accelerated the spread of the disease and provided optimal conditions for the growth of the fungus, affecting more and more hosts. That is why more research is needed to optimize the synergies of these green alternatives that allow scaling, industrial production, and commercial testing of these bioproducts so that exporters, industries, and farmers can adopt them. In the future, substances of natural origin and antagonistic microorganisms could replace the chemicals currently used to protect fruits.

## Figures and Tables

**Table 1:** Different studies applying essential oils for in vitro and in vivo control of *C. gloeosporioides*.

Essential Oil	Fruit/Crop In Situ Studies	Extraction Method	Evaluation Type	Minimum Inhibition Concentration	References
Mexican oregan oil (*Poliomintha longiflora*)	-	Steam distillation	In vitro	1.0 (g/L)	[53]
Rosemary (*Rosmarinus officinalis*) Mint (*Mentha piperita*) Lemongrass (*Cymbopogon citratus*) Anise (*Pimpinella anisum*) Tea tree (*Melaleuca alternifolia*) Cinnamon (*Cinnamomum zeylanicum*)	Papaya	N. M. commercial samples	In vitro and In situ	100 (µL/mL)	[54]
Salvia oil (*Salvia officinalis*) Rosemary oil (*Rosmarinus officinalis*) Oregano oil (*Origanum vulgare*) *Eucalyptus* sp. Fennel oil (*Foeniculum vulgare*)	Golden apples	Steam distillation	In vitro and In situ	N.D.*	[55]
Thyme (*Thymus vulgaris*) Clove (*Syzygium aromaticum*) Cinnamon (*Cinnamomum zeylanicum*)	Mango	N. M. commercial samples	In vitro and In vivo	66.7 (µL/L)	[56]
Mint oil (*Mentha piperita* Willd.) Savory oil (*Satureja khuzistanica* Jamzad.) Thyme oil (*Thymus daenensis* Celak.) Cinnamon oil (*Cinnamomum zeylanicum* Blume.) Lavender oil (*Lavandula angustifolia* Mill.)	Avocado	Hydrodistillation	In vitro and In situ	2000 (µL/L)	[57]
Lemongrass oil (*Cymbopogon citratus*)	-	Microwave-assisted hydrodistillation (MWHD)	In vitro	10,000 (µg/L)	[58]
Seventy-five oils. *Cinnamomum cassia*, *Cinnamomum zeylanicum*, *Crocus sativus,* and *Syzygium aromaticum* oils were the most effective.	-	N. M. commercial samples	In vitro	5 µL (pure)	[59]
*Corymbia citriodora* *Cymbopogon citratus* *Cymbopogon flexuosus* *Curcuma longa*	-	Hydrodistillation	In vitro	10–20 µL (pure)	[60]
Rosewood oil (*Aniba duckei* Kostermans)	-	Hydrodistillation	In vitro	2000 (µL/mL)	[61]
Swingle *oil (Murraya microphylla)* (Merr.)	-	Hydrodistillation (Column chromatography with silica gel)	In vitro	63.3 µL (ED_50_) crude oil (3.24 µL for Terpinen-4-ol)	[62]
Basil (*Ocimum basilicum*) Citronella (*Cymbopogon winterianus*) Clove (*Syzygium aromaticum*) Copaiba (*Copaifera langsdorfii*) Eucalyptus (*Eucalyptus citriodora*) Mint (*Mentha arvensis*) Rosemary (*Rosmarinus officinalis*) Tea tree (*Melaleuca alternifolia*)	Pepper seeds	N. M. commercial samples	In vitro and In situ	2500 (µg/mL)	[63]
Cardamom (*Elettaria cardamomum*) Citronella (*Cymbopogon nardus* L.) Lemon (*Citrus limon*) Mustard (*Brassica juncea* L.) Orange (*Citrus sinensis*)	-	N. M. commercial samples	In vitro	750–1000 (µL/L)	[64]
*Ruta graveolens* essential oil	Papaya	N. M. commercial samples	In situ	N.D. *	[65]

* N.D.: Not determined.

**Table 2:** Different studies applying chitosan–essential-oils (nano)composites for in vitro and in vivo control of *C. gloeosporioides*.

Chitosan System	Food	Essential Oil Concentration	Results	References
Chitosan + *Ruta graveolens* essential oil (Chi+REO)	Guava (*Psidium guajava* L.)	500–1500 μg/mL	Chi + REO coated fruit had less antimicrobial decay without affecting the organoleptic properties. Chi + REO (1500 μg/mL) coatings managed to reduce the area of infection in guavas by up to 70%. Chi + REO coated fruits were stable and accepted by consumers for up to 12 days.	[83]
Chitosan + *Ruta graveolens* essential oil (Chi+REO)	Papaya (*Carica papaya* L.)	500–1500 μg/mL	Chi + REO (1000 and 1500 μg/mL) coated papaya were stable and accepted by consumers for up to 12 days. The coated papaya did not present negatively altered organoleptic characteristics. The fungus treated with Chi + REO showed ultrastructural cell and conidia damage, with a 90% reduction in the conidia germination.	[29]
Chitosan and *Mentha x villosa* Huds or *M. piperita* L. essential oil (Chi+MVEO) (Chi+MPEO)	Papaya (*Carica papaya* L.)	Chi: 5 g/L MVEO: 0.6, 1.2 mL/L MPEO: 0.6, 1.2 mL/L	Chi + MVEO or MPEO coatings showed interactions and molecular compatibility from the analysis of the homogeneous microstructure and the improvements in thermal stability. The coated fruits showed less ripening during the cold storage time, and minor changes in physicochemical properties were evaluated. Coatings did not affect consumer acceptability.	[84]
Chitosan and *Cymbopogon citratus* (D.C.) Stapf. (Chi+CCEO)	Guava (*Psidium guajava* L.)	0.6 μL/mL	Chi + CCEO coatings inhibited the affectation of *C. gloeosporioides* in the coated guavas and improved the physicochemical properties evaluated.	[85]
Chitosan + *Ruta graveolens* essential oil (Chi+REO)	Papaya (*Carica papaya* L.)	500 μg/mL	The Chi+ REO emulsions after nine days of storage at 25 °C reduced the anthracnose incidence by 37%, with disease severity and McKinney’s decay index decreased by 44%. The Chi+ REO treatment also demonstrated additive actions on gene expression in triggering defense pathways linked to the fruit.	[65]

## Data Availability

Not applicable.
